# Designing Patient-Driven, Tissue-Engineered Models of Primary and Metastatic Breast Cancer

**DOI:** 10.3390/bioengineering9020044

**Published:** 2022-01-18

**Authors:** Garrett F. Beeghly, Candace Thomas, Jessica X. Yuan, Alexandra R. Harris, Jennifer M. Munson

**Affiliations:** 1 Department of Biomedical Engineering, University of Virginia, Charlottesville, VA 22904, USA; cft2ct@virginia.edu (C.T.); jxy9gw@virginia.edu (J.X.Y.); arh5ru@virginia.edu (A.R.H.); 2 Fralin Biomedical Research Institute, Virginia Polytechnic and State University, Roanoke, VA 24016, USA

**Keywords:** breast cancer metastasis, tumor microenvironment, fibroblasts, astrocytes, collagen, hyaluronan, matrix remodeling

## Abstract

The rising survival rate for early-stage breast cancer in the United States has created an expanding population of women in remission at risk for distant recurrence, with metastatic spread to the brain demonstrating an especially poor prognosis. The current standard of care for breast cancer brain metastases is not well defined or differentiated from the treatment of brain metastases from other primary sites. Here, we present tissue-engineered models of the primary and brain metastatic breast cancer microenvironments informed by analysis of patient tumor resections. We find that metastatic resections demonstrate distinct cellular and matrix components compared with primary resections or non-cancerous controls. Using our model systems, we find that the observed deposition of collagen I after metastasis to the brain may enhance breast cancer invasion. Future optimization of these models will present a novel platform to examine tumor-stroma interactions and screen therapeutics for the management of metastatic breast cancer.

## 1. Introduction

One significant achievement of modern medicine is the extent of public and institutional support afforded to women with breast cancer in the United States. The five-year survival rate for women with breast cancer has improved over the past three decades, rising to 91% on average and 99% for patients with early-stage disease due to improved screening and advances in treatment [1]. However, this continuous increase in survival rate has led to a surge in the number of breast cancer survivors living in the United States, with the figure currently estimated at 3.9 million women. These patients, considered to be in remission, demonstrate high risk for distant recurrence or metastasis. Indeed, of these 3.9 million women, up to 24% are expected to develop metastases to the brain during their lifetimes [2]. Distant recurrence to the brain is associated with the shortest survival time and poorest prognosis compared with other sites of spread, with the time from diagnosis until death taking just 17 months on average. Given this rapidly expanding high-risk population, the current standard of care for the management of metastatic breast cancer needs to be reassessed in order to meet the needs of breast cancer survivors living in remission.

The current standard of care for breast cancer brain metastases is not well defined and includes whole-brain radiation therapy and surgical resection if possible [3]. However, whole-brain radiation therapy has not been shown to have a significant impact on patient survival and can result in severe side effects such as memory loss, exhaustion, and dementia. In addition, recurrence after resection is common given that surgery has a conservative nature in order to minimize the removal of healthy brain tissue. Furthermore, all brain metastases receive the same treatment regardless of the original tumor type, indicating a need to better understand how unique microenvironmental interactions within the brain affect cancer progression [3]. Despite these shortcomings, each breast cancer patient diagnosed with brain metastases generates almost $20,000 per month on average in healthcare-related expenses [4]. Given the limited therapeutic and economic efficacy of these options, the creation of improved treatments will be essential for the future management of breast cancer brain metastases.

Increasing evidence implicates the tumor microenvironment, or the tissue adjacent to the tumor bulk known as the stroma, in regulating cancer cell behavior [5,6]. This microenvironment is an inherently complex system consisting of stromal cells such as fibroblasts and immune cells, blood and lymphatic vessels, and biophysical forces such as interstitial fluid flow. These components have been shown to aid malignant progression and contribute to therapeutic resistance across tumor types [6,7]. In particular, resident fibroblasts within the breast are common stromal cells associated with enhanced tumor invasion [8,9,10]. Astrocytes demonstrate similar effects for gliomas within the brain [11]. Although both these stromal populations are known to mediate matrix remodeling under physiological conditions in the breast and brain, respectively, the molecular mechanisms by which they promote invasion in the context of cancer are less clear. Understanding the differences between how these components modulate breast cancer behavior will serve as an initial step to inform the creation and modification of therapies for the management of breast cancer after metastasis to the brain. Three-dimensional multicellular and matrix models are an established approach for investigating the relationships between tumor cells and their microenvironments [12,13,14]. Here, we present models of primary and metastatic breast cancer based on immunofluorescence of patient tumor resections. These tissue-engineered models serve as vehicles to examine tumor-stroma interactions in vitro and screen therapeutics for the management of breast cancer brain metastases.

## 2. Materials and Methods

### 2.1. Sample Selection

Patient samples were accessed through the University of Virginia Biorepository and Tissue Research Facility. These samples were selected from archived patients with a definitive diagnosis of breast cancer who received no treatment prior to tumor resection. Samples were de-identified before use. All procedures performed in studies involving human participants were in accordance with the ethical standards of the institutional review board of the University of Virginia and with the 1964 Helsinki declaration and its later amendments or comparable ethical standards.

### 2.2. Immunofluorescence

Formalin-fixed, paraffin-embedded sections were deparaffinized with xylene and rehydrated in graded ethanol solutions. Antigen retrieval was performed by boiling the samples for 30 min in a citrate-based antigen unmasking solution (Vector Labs, Burlingame, CA, USA). The samples were washed two times with permeabilization solution (0.1% Triton-X in 1× TBS) and then incubated at room temperature with blocking solution based on secondary antibody hosts (4% serum in permeabilization solution). The primary antibodies were diluted in blocking solution and applied to the samples overnight at 4 °C. The samples were then washed three times with permeabilization solution and incubated at room temperature with secondary antibodies diluted in 2% bovine serum albumin in permeabilization solution. The samples were incubated with DAPI (Sigma-Aldrich, St. Louis, MO, USA) diluted to 1.43 μM in 1× TBS and washed three times with 1× TBS. Residual liquid was removed from the slides before the samples were mounted with Fluoromount-G (SouthernBiotech, Birmingham, AL, USA) and sealed with nail polish. The following reagents were used for immunofluorescence: anti-alpha-smooth muscle actin (Thermo Scientific, Waltham, MA, USA, 41-9760-80, 1 μg/mL), anti-pan-keratin (Thermo Scientific, Waltham, MA, USA, MS-343-P, 4 μg/mL), anti-GFAP (Abcam, Cambridge, UK, 7260, 1:1000 dilution), anti-collagen I (Rockland, Pottstown, PA, USA, 34755, 1:200 dilution), anti-tenascin C (R&D, Minneapolis, MN, USA, AF3358, 1 μg/mL), hyaluronic acid binding protein (Millipore, Burlington, MA, USA, 385911, 2.5 μg/mL).

### 2.3. Image Analysis

Stained slides were analyzed with an EVOS fluorescent cell imaging system (Thermo Scientific, Waltham, MA, USA) and processed using ImageJ (National Institutes of Health, Bethesda, MD, USA). Random non-overlapping 856 × 476 µm (407,465 µm^2^) regions within the tumor or stroma were selected for imaging. The number of regions analyzed varied depending on the size of the tissue sample. Based on markers previously established in the literature, breast cancer cells were identified by anti-pan-cytokeratin staining, cancer-associated fibroblasts were identified by anti-alpha-smooth muscle actin staining, and activated astrocytes were identified by anti-GFAP staining [15,16,17]. In ImageJ, the “Merge Channels” command was used to false color and overlay individual images. Confocal imaging was performed on a Zeiss LSM 700 microscope running ZEN 2009 software (Zeiss, Oberkochen, Germany).

### 2.4. Cell Culture and Labeling

The human breast cancer cell line MDA-MB-231 and human dermal fibroblasts were obtained from ATCC (Manassas, VA, USA) and human cortical astrocytes were obtained from Sciencell (Carlsbad, CA, USA). MDA-MB-231 cells and fibroblasts were cultured and maintained in Dulbecco’s Modified Eagle’s Medium (DMEM; Life Technologies, Carlsbad, CA, USA) supplemented with 10% FBS. Astrocytes were cultured in supplemented astrocyte medium (Sciencell, Carlsbad, CA, USA). Cells were labeled with fluorescent CellTracker Dyes (Thermo Scientific, Waltham, MA, USA) at recommended dilutions in serum-free media for one hour before use. 

### 2.5. Preparation of Tissue-Engineered Models

Experiments were carried out with 12 mm-diameter, 8 μm-pore tissue-culture inserts (Millipore). Hydrogels were created based on previous publications [12,18,19]. For collagen-based gels, 1.8 mg/mL rat tail collagen I (Corning, Corning, NY, USA), 0.2 mg/mL basement membrane extract (Trevigen, Gaithersburg, MD, USA), serum-free DMEM, 10× PBS, 1 M NaOH, and distilled water were combined in a microcentrifuge tube before the addition of labeled cells. For hyaluronan-based gels, 1.2 mg/mL thiolated hyaluronan (Glycosil; ESI BIO, Alameda, CA, USA), 1.2 mg/mL rat tail collagen I, serum-free DMEM, 10× PBS, 1 M NaOH, distilled water, and Extralink PEDGA (ESI BIO, Alameda, CA, USA) were combined in a microcentrifuge tube before the addition of labeled cells. Gel volume was kept constant at 100 μL and cell densities determined from immunofluorescence were normalized to 120,000 cells per gel and below. 700 μL of serum-free DMEM was placed outside of the insert and 100 μL were placed inside the insert for standard static conditions. These volumes were switched to create a pressure gradient for flow conditions. This pressure head results in linear flow velocities between 0.7–1.2 µm/s. Gels were analyzed after 18–24 h of incubation. Experiments without invasion as an outcome measure were completed in 96-well plates with a reduced gel volume of 50 μL and densities of 60,000 cells per gel and below to conserve reagents.

### 2.6. Invasion Assays and Quantification

After 18 h, gels were removed and the number of MDA-MB-231 cells remaining on the underside of the insert membrane were counted as a relative measure of tumor cell invasion. Five random fields of view were imaged and counted per insert to extrapolate the total percentage of seeded cells that had migrated through the membrane. Percent invasion was calculated as the number of cells that traversed the membrane/number of cells seeded × 100.

## 3. Results

### 3.1. Immunofluorescence of Primary and Metastatic Breast Cancer Resections

**Figure 1:**
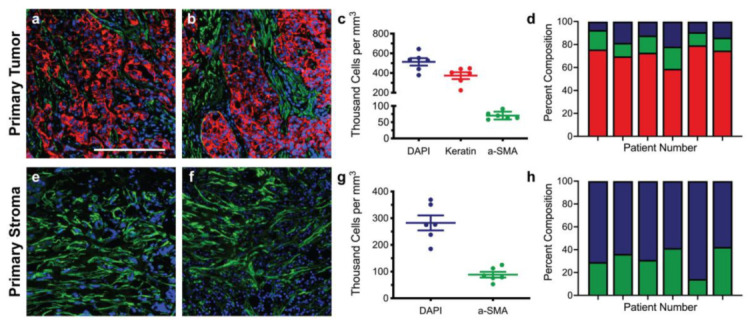
Staining and cellular quantification of primary breast cancer resections. Primary breast cancer patient resections were stained for breast cancer cells (anti-pan keratin; red), activated fibroblasts (anti-αSMA; green), and total cells (DAPI; blue). The number of positively stained cells for each channel was counted in five random regions in order to determine the cell densities and percent compositions within the tumor (**a**–**d**) and within the stroma (**e**–**h**) for six patients (*n* = 6). Scale bar = 200 μm.

**Figure 2:**
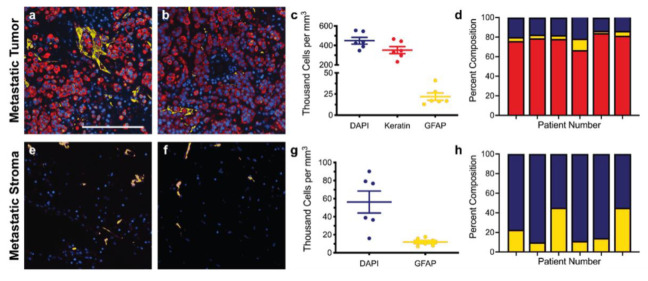
Staining and cellular quantification of metastatic breast cancer resections. Breast cancer brain metastases patient resections were stained for breast cancer cells (anti-pan keratin; red), activated astrocytes (anti-GFAP; yellow), and total cells (DAPI; blue). The number of positively stained cells for each channel was counted in five random regions in order to determine the cell densities and percent compositions within the tumor (**a**–**d**) and within the stroma (**e**–**h**) for six patients (*n* = 6). Scale bar = 200 μm.

**Figure 3:**
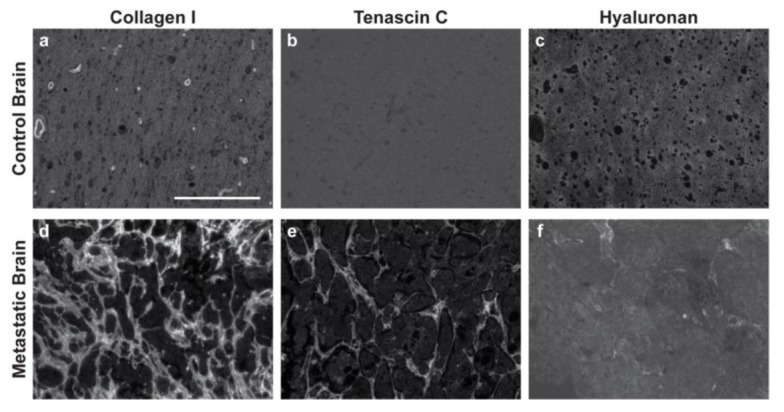
Breast cancer brain metastases exhibit remodeled extracellular matrix. Non-cancerous control brain resections (**a**–**c**) and breast cancer brain metastases resections (**d**–**f**) were stained for collagen I (anti-collagen I), tenascin C (anti-tenascin C), and hyaluronan (hyaluronic acid binding protein). Representative images were selected across six patients (*n* = 6). Scale bar = 200 μm.

Immunofluorescence of both primary and metastatic triple-negative breast cancer patient resections was performed to establish appropriate cell seeding ratios and matrix compositions for the respective tissue-engineered models. Primary resections were stained for breast cancer cells (anti-pan keratin) and activated fibroblasts (anti-αSMA) and then regions of the tumor (Figure 1a,b) and stroma (Figure 1e,f) were quantified to determine the cell densities across six patient samples. Metastatic resections were stained for breast cancer cells (anti-pan keratin) and activated astrocytes (anti-GFAP) and then regions of the tumor (Figure 2a,b) and stroma (Figure 2e,f) were similarly quantified to determine the cell densities across six separate patient samples. Five distinct regions of the tumor and five distinct regions of the stroma were analyzed per patient. In general, primary and metastatic resections demonstrated similar total cell densities within the tumor, around 500,000 cells per mm^3^ (Figure 1c and Figure 2c). In contrast, the cell densities of the stroma surrounding the primary tumors were more than twice as dense as the corresponding stroma in the brain (Figure 1g and Figure 2g). Moreover, astrocytes were not as prevalent within the metastatic tumor bulk and stroma, exhibiting densities about one order of magnitude lower in both locations than fibroblasts exhibited in the breast. However, for both primary (Figure 1d,h) and metastatic (Figure 2d,h) resections, the cellular composition (as a percentage of total cells) demonstrated a fair degree of heterogeneity between patients. Metastatic resections were also stained for the matrix components collagen I (anti-collagen I), tenascin C (anti-tenascin C), and hyaluronan (hyaluronic acid binding protein). While hyaluronan remained consistently diffuse between metastatic and non-cancerous control resections (Figure 3c,f), the metastatic resections demonstrated unexpected networks of fibrillar collagen I and tenascin C (Figure 3a,b,d,e). In contrast, the non-cancerous control resections only demonstrated positive staining for collagen I around blood vessels, as expected for healthy brain tissue [20]. Overall, immunofluorescence revealed that primary fibroblast densities were significantly greater than metastatic astrocyte densities (Figure 1g and Figure 2g) and that the metastatic tumor microenvironment contains more fibrillar collagen I and tenascin C than non-cancerous controls (Figure 3).

### 3.2. Design and Composition of Representative Tissue-Engineered Models

**Figure 4:**
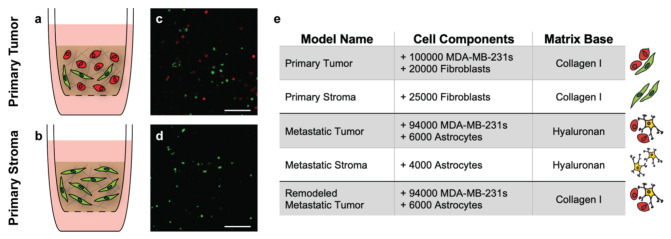
Tissue-engineered model parameters. Graphical depictions of the primary tumor and stroma models indicate the cellular and matrix components within each (**a**,**b**). Representative cross-sectional images of the models 18 h after formation show successful incorporation of labeled fibroblasts (green) and MDA-MB-231 breast cancer cells (red) (**c**,**d**). Scale bars = 200 μm. Summary of the cell components and matrix base for each of the corresponding tissue-engineered models (**e**).

Previously validated hydrogel systems were selected as the base for our models of the primary and metastatic breast cancer microenvironments. A collagen-based gel was selected for the primary model given that breast tissue is rich in fibrillar collagen I, while a hyaluronan-based gel was selected for the metastatic model as hyaluronan is an essential component of brain tissue which contains far fewer fibrillar proteins [12,13]. However, to account for the extensive, unexpected presence of fibrillar collagen I observed in metastatic patient resections (Figure 3d), we also established an additional “remodeled” metastatic tumor condition where the metastatic cell components were seeded in a collagen-based gel. We normalized the cell densities determined by immunofluorescence for incorporation into the models such that the highest density was 120,000 cells per gel (primary tumor) and all other densities were proportionally lower. Graphical depictions of the primary tumor and stroma models, along with corresponding cross-sectional images of each are illustrated in Figure 4a–d. The cell components and gel-base for all models are enumerated in Figure 4e. Note that the incorporated cell populations were fluorescently labeled before use. A breakdown of the collagen-based and hyaluronan-based gel compositions can be found in the methods section.

### 3.3. The Tumor Microenvironment Affects Breast Cancer Invasion

**Figure 5:**
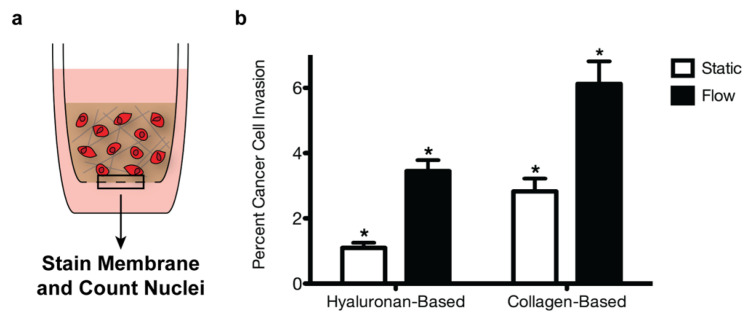
Extracellular matrix composition and fluid flow affect breast cancer cell invasion. MDA-MB-231 breast cancer cells were seeded into collagen-based or hyaluronan-based gels within tissue-culture inserts (**a**). After 18 h, percent invasion was calculated by dividing the number of cells in the insert membrane by the number of seeded cells (**b**). A two-way ANOVA and Bonferroni comparison test were performed to determine statistical significance (* *p* < 0.05). Error bars represent standard deviation (*n* = 3).

In order to examine the effect of the collagen I deposited in the metastatic brain resections on breast cancer cell invasion, we seeded MDA-MB-231 breast cancer cells into collagen-based or hyaluronan-based gels polymerized in tissue-culture inserts. We selected the highly invasive, triple-negative MDA-MB-231 cell line for these studies as triple-negative breast cancer patients with brain metastases demonstrate the poorest prognosis compared to other molecular subtypes [21,22]. In addition, gels were cultured under either static or flow conditions to mimic interstitial fluid flow, which is known to enhance breast cancer cell invasion [13]. For flow conditions, a media head was added on top of the gels to generate pressure-driven fluid flow on the order of 1 μm/s, similar to rates observed in vivo [12,13]. After 18 h, the number of MDA-MB-231 breast cancer cells in the tissue-culture insert membranes was quantified as a relative measure of invasion. Significantly more cells invaded through the collagen-based gels than the hyaluronan-based gels under flow conditions (Figure 5). Moreover, a similar increase was observed for the static conditions, indicating that a change in the matrix composition of the culture environment alone affected breast cancer cell motility.

### 3.4. Creation of Multi-Layered Models to Mimic the Tumor-Stroma Interface

**Figure 6:**
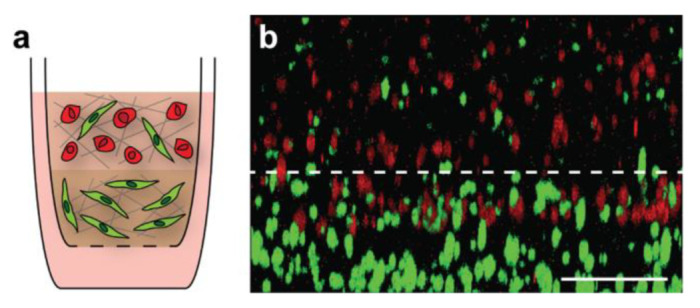
Layering of primary tumor and primary stroma models. Schematic of multi-layered breast cancer model (**a**). MDA-MB-231 breast cancer cells were labeled with CellTracker Deep Red and fibroblasts were labeled with CellTracker Green prior to use. A three-dimensional image was obtained of the boundary between the top tumor model and bottom stroma model 18 h after gel formation (**b**). Gel orientation remained intact after fixation and removal from the tissue-culture insert. Breast cancer cell invasion from the tumor layer into the stroma layer can be seen, indicating that cells were able to migrate through the gel boundary. Scale bar = 200 μm.

To further examine how breast cancer cells interact with the surrounding microenvironment, we layered our tumor and stroma models (Figure 6a) to recapitulate the transition zone found at the tumor-stroma border in vivo as a proof of concept. These stacked gels allowed for regions of differing cell densities and matrix components to be included within the same tissue-culture insert for analysis. We were able to optimize the layering process for our primary tumor and stroma models, ensuring that there was a distinct boundary between the gels while maintaining the ability of MDA-MB-231 breast cancer cells to migrate into the lower stromal layer within 18 h of gel formation (Figure 6b).

## 4. Discussion

Here, we present patient-driven, tissue-engineered models of primary and metastatic breast cancer. From immunofluorescence of tissue resections, we observe differences in the cellular and matrix composition between primary and metastatic tumors, as well as between individual patients. Using these models, we found that both matrix composition and the addition of fluid flow influence breast cancer invasion. Breast cancer cells use integrin heterodimers and CD44 to migrate by engaging with collagen I and hyaluronan, respectively [23,24]. The greater invasion rate in collagen-based gels suggests that integrin-based migration may be more efficient for these cells if available, though the study is limited by the use of a single representative cell line. While differences in substrate mechanics could also influence tumor cell migration, the collagen- and hyaluronan-based gels used here have similar elastic moduli on the order of 1000 Pa [12]. Of note, high concentrations of collagen I are correlated with a poor prognosis and predisposition for metastasis in primary breast cancer [25]. The extensive collagen I deposition we observed in the metastatic patient resections presents a distinct matrix phenotype from healthy brain tissue and primary brain tumors, which may worsen clinical outcomes. Collectively, these results suggest that breast cancer cells mediate deposition of fibrillar collagen I in the otherwise nanoporous hyaluronan-based brain extracellular matrix to promote metastatic progression. This elevated degree of collagen I in the brain could be secreted by metastatic breast cancer cells [26], by recruited progenitor cells [27], or by astrocytes or other neuroglial cells induced to secrete collagen I in response to tumor-derived signals [28,29]. Therefore, future work is needed to understand what molecular mechanisms guide this matrix remodeling and if these factors could be targeted therapeutically.

Multicellular and matrix models offer many advantages over traditional 2D co-culture systems for examining tumor-stroma interactions and response to treatment. For example, these platforms provide a 3D culture environment, which has been shown to regulate tumor cell behavior ranging from migration to chemoresistance, and include matrix ligands to better mimic human disease [30,31]. However, several limitations prevent these models from fully recapitulating the microenvironment found in vivo. The use of previously validated hydrogels made of natural biomaterials does not allow for the material properties of each condition to be independently tuned. For example, each collagen-based gel contained the same concentration of collagen I and therefore did not account for documented differences in matrix density, ligand presentation, and tissue stiffness observed between regions of tumor and stroma [32,33]. Moreover, these models cannot be used to culture cells at the densities found within human tumors while maintaining cell viability and structural integrity. Despite these limitations, the presented models offer a novel approach for examining breast cancer behavior and screening therapies in a tractable setting prior to animal studies and clinical trials. In the future, the described model systems can be adapted to investigate other tumor-stroma interactions that mediate cancer progression, with the end goal of improving treatment options for patients with metastatic breast cancer.

## Data Availability

Data available on request due to IRB restrictions.
